# Bovine dermatophilosis: Awareness, perceptions and attitudes in the small-holder sector of north-west Zimbabwe

**DOI:** 10.4102/ojvr.v83i1.1004

**Published:** 2016-03-09

**Authors:** Daud N. Ndhlovu, Patrick J. Masika

**Affiliations:** 1 Department of Clinical Veterinary Studies, University of Zimbabwe, Zimbabwe; 2 Fort Cox College of Agriculture and Forestry, Cwaru, South Africa

## Abstract

A cross-sectional study was conducted to assess cattle owners’ awareness, perceptions, attitudes and drug-usage practices with regard to bovine dermatophilosis. Knowledge of these farmers’ attributes is important for animal health policy makers in their endeavours to provide optimum disease control strategies that are acceptable to the communities. Data on cattle owner awareness of bovine dermatophilosis, causes, treatment practices, perceptions about its importance and potential dangers to humans were collected using an interviewer-administered questionnaire. A total of 185 stockowners and cattle herds were involved in the study, with bovine dermatophilosis determined clinically by veterinarians. The results showed that 45.4% of the herds were clinically positive for dermatophilosis, and most farmers (79.5%) were generally aware that dermatophilosis was a cattle disease. In the event of a dermatophilosis outbreak in a herd, 74.1% of the farmers treated their cattle using antibiotics; the proportion of farmers treating cattle did not differ (*p* > 0.05) across the diptanks. Fifty-two farmers (52/63) indicated that drugs had to be administered four to seven times before an animal recovered from infection. Tetracyclines were the antibiotics used by most farmers (79.3%) to treat dermatophilosis, with 19.1% using penicillins. Concerns were raised by farmers about the effectiveness of these drugs against bovine dermatophilosis. Across the study sites, 48.6% and 27.6% of the farmers perceived bovine dermatophilosis to be an important disease at the herd and area level, respectively. A small proportion (12.4%) of the farmers regarded bovine dermatophilosis as a potentially zoonotic disease. The high level of stockowners’ general awareness, with regards to bovine dermatophilosis, sets ideal conditions for the mobilisation of farmers by animal health authorities in the control of the disease. However, further research needs to be undertaken to investigate effective antibiotic delivery protocols and the potential zoonotic impact of bovine dermatophilosis in a situation of high disease prevalence.

## Introduction

Bovine dermatophilosis is an important disease of cattle in Africa, it was first reported in the Democratic Republic of Congo (then Belgian Congo) in 1915 (Oppong 1996) and has been reported in most countries in the African continent (Hamid & Musa 2009), including Zimbabwe (Chatikobo *et al*. 2009; Ndhlovu & Masika 2015). Bovine dermatophilosis is a tick-associated disease caused by an actinomycete bacterium, *Dermatophilus congolensis* (Blood & Radostits 1989; Molia *et al*. 2008), characterised by an exudative acute or chronic dermatitis that could be localised or generalised (Admassu & Alemu 2011). The lesions vary in severity, from small lesions (small paint brush-like) and clear circumscribed scabs to more confluent progressive lesions (Hadrill & Walker 1996). Stewart (1972) described a carrier status in cattle, in which the lesions were not easily observed and it was concluded that carrier animals were the principal means of survival for *D. congolensis*. The disease can occur in tick-free animals, but it is more severe in those that are infested by *Amblyomma variegatum* ticks (Stachurski, Zoungrana & Konkobo 2010). Walker (1996) stated that the role of *A. variegatum* in the development of dermatophilosis was through immunosuppression, a fact further supported by Estrada-Peňa *et al*. (2007). The tick was postulated to secrete an immunosuppressive agent in its saliva or waste metabolites that were toxic to the host. Other factors that predispose to the disease are wetting of the skin and trauma (Hirsh, Maclachlan & Walker 2004). Economically, bovine dermatophilosis is important because of morbidity and mortality, damage to hides and its effect on draught animal power (Ahoussou *et al*. 2010; Bayisa *et al*. 2012). In other parts of Africa, it has frustrated the introduction of exotic breeds to improve meat and milk production (Koney 1996).

The treatment of bovine dermatophilosis is mainly by the use of penicillin, streptomycin and dihydrostreptomycin given intramuscularly (Hamid & Musa 2009). Awad, Nadra-Elwgoud and El-Sayed (2008) indicated that a double dose of long-acting tetracyclines given a day apart gave better results than a single dose. A combination of penicillin and streptomycin has been reported to produce a cure, whilst gentamycin was reportedly the most effective antibiotic (Hamid & Musa 2009). Acaricides have been reported to be the best option for the control of bovine dermatophilosis (Hadrill & Walker 1996). Amitraz-based acaricides as well as deltamethrin applied at the predilection sites of *A. variegatum* ticks on cattle reduced the prevalence of dermatophilosis (Morrow *et al*. 1993; Morrow, Koney & Heron 1996). The method of tick control is important in the management of bovine dermatophilosis. However, Chatikobo *et al*. (2001) reported that plunge dipping could in fact increase the risk of spread of the disease, whilst hand spraying reduced the risk of spread.

In Zimbabwe, bovine dermatophilosis has become a disease of importance to small-holder farmers. As a result of its spread to previously uninfected areas (Chatikobo *et al*. 2009), the Department of Livestock and Veterinary Services (DLVS) has developed two statutory instruments (SI) to aid in its control (Government of Zimbabwe 2010a, 2010b). Statutory Instrument 166 of 2010 (Animal Health [Dermatophilosis Areas] Order 2010) (Government of Zimbabwe 2010a) defines certain districts in the country as dermatophilosis-prone areas. The Statutory Instrument 167 of 2010 (Animal Health [Dermatophilosis] Regulations 2010) (Government of Zimbabwe 2010b) regulates what the farmers should do and lists the authorised persons to reach out for the control of dermatophilosis. To augment existing control measures, research has also been conducted focusing on certain aspects of the control, prevalence and distribution of bovine dermatophilosis (Chatikobo *et al*. 2001, 2004, 2009; Ndhlovu & Masika 2013, 2015). However, there is a paucity of information about the farmers’ perceptions, attitudes and usage of drugs with regard to the disease. Because the cooperation of famers will be necessary to achieve a good level of implementation of control measures prescribed by the authorities, knowledge of these attitudes, perceptions and practices will assist animal health decision makers in developing optimum control and management strategies for bovine dermatophilosis.

The current study was designed to determine the general awareness, perceptions, attitudes and the drug-usage practices with regard to bovine dermatophilosis amongst small-holder farmers from selected diptanks in central and north-west Zimbabwe.

## Materials and methods

### Study sites

The study was carried out at four small-holder diptanks located in the central and north-west areas of Zimbabwe, these being Chivero (-18° 21’S: 30° 36’E), Koronika (-18° 07’S: 29° 26’E), Gwanyika (-18° 24’S: 29° 12’E) and Chemawororo (-18° 19’S: 28° 47’E). Chivero diptank is located in agro-ecological region 3. Region 3 is characterised by semi-intensive farming, moderate rainfall (650 mm – 800 mm) and a mean annual temperature range of 18 °C – 22 °C; the other three diptanks were located in region 4, characterised by semi-extensive farming, moderate to erratic rainfall (650 mm – 800 mm) and a mean annual temperature range of 18 °C – 24 °C (Chikodzi *et al*. 2013; Mugandani *et al*. 2012).

### Study design and questionnaire survey

A cross-sectional study was carried out from September 2013 to April 2014 to determine the awareness, perceptions, attitudes and drug-usage options of farmers with regard to bovine dermatophilosis. A minimum of 29 and a maximum of 60 stockowners were systematically selected (Dohoo, Martin & Stryhn 2003) from each of the four diptanks. The systematic sampling was conducted by sampling every fifth herd that was about to enter the diptank race; the estimated number of herds per diptank was 200–300 (personal observation). A pretested structured questionnaire was administered to a total of 185 stockowners who presented 185 cattle herds with a total of 1788 cattle. The questionnaire was divided into six main sections: (1) general information, (2) cattle demography, (3) history and knowledge of bovine dermatophilosis, (4) current cases of bovine dermatophilosis and (5) management of dermatophilosis (i.e. drugs administered, frequency). Because there were a number of diseases that could be mistaken for bovine dermatophilosis, for example, lumpy skin disease (LSD), respondents were further asked to describe how they differentiated this disease from the others. The presence or absence of current bovine dermatophilosis cases was clinically determined by physically examining the cattle. The case definition for clinical dermatophilosis was as described by Hadrill and Walker (1996) and the clinical cases were confirmed by the veterinarians including the principal investigator (Ndhlovu & Masika 2015). Stockowners were interviewed separately by the principal investigator and the two district veterinarians, with interviews being conducted in the local Shona and isiNdebele languages.

## Data analysis

Questionnaire data from the field were entered in EpiInfo™ version 7.1.4.0 (2014). Data were analysed using STATA/ SE 11.2 (StataCorp LP 2012) to generate descriptive statistics (frequencies/proportions) related to cattle owners’ awareness/perceptions of bovine dermatophilosis such as knowledge of the causes, treatment/control and clinical signs of dermatophilosis, whether dermatophilosis was a danger to humans and the drugs used to treat the disease, frequency of use and effectiveness. Fisher’s exact test was used to evaluate associations between categories, whilst the two-sample proportion test calculator and the Marascuillo procedure in Microsoft Excel (^XLS^ChiSquare2x3; www.stat.ufl.edu/_~_mrpol/QBM/…/Chi-Square%worksheets.xls) was used to compare column proportions, respectively. Values of *p* < 0.05 were considered as significant.

## Results

Eighty-four (45.4%) stockowners interviewed had at least one herd of cattle that had clinical bovine dermatophilosis diagnosed by a veterinarian. In the event that cattle were affected with the disease, 63% (overall, 34.1%) or 74.1% of the affected stockowners treated their cattle (Table 1). The number of farmers who treated cattle for dermatophilosis differed (*p* < 0.05) in the category that treated only (Chemawororo differed), whilst in the category that did not treat, Chivero differed (Table 2).

**TABLE 1:** Number of respondents, herds affected and farmers who treat for dermatophilosis.

Diptank	Farmers/cattle-herds	Cases		Treated	
	
		*n*	%	*n*	%
Chemawororo	60	34	56.7	25	41.7
Chivero	29	2	6.9	2	6.9
Gwanyika	52	25	48.1	22	42.3
Koronoka	44	23	52.3	14	31.8

**Total**	**185**	**84**	**45.4**	**63**	**34.1**

**TABLE 2:** Stockowners’ knowledge and perceptions about bovine dermatophilosis according to diptank.

Factor	Level	Chemawororo		Chivero		Gwanyika		Koronika		Total	
				
		*n*	%	*n*	%	*n*	%	*n*	%	*n*	%
Heard of dermatophilosis	Yes	59^a^	98.3	4^b^	13.8	47^a^	90.4	37^a^	84.1	147	79.5
	No	1^a^	1.7	25^b^	86.2	5^ac^	9.6	7^c^	15.9	38	20.5
Aware of cause(s)	Yes	47^a^	78.3	0^b^	0.0	39^a^	75.0	9^c^	20.5	95	51.4
	No	13^a^	21.7	29^b^	100.0	13^a^	25.0	35^c^	79.5	90	48.6
Aware of treatment	Yes	39^a^	65.0	0^b^	0.0	29^a^	55.8	17^a^	38.6	85	45.9
	No	21^a^	35.0	29^b^	100.0	23^ac^	44.2	27^c^	61.4	100	54.1
Local name	Do not Know (D/K)	1^a^	1.7	26^b^	89.7	5^a^	9.6	9^c^	20.5	41	22.2
	*Chikundura*	59^a^	98.3	0^b^	0.0	47^a^	90.4	33^c^	75.0	139	75.1
	Senkobo	0^a^	0.0	3^b^	10.3	0^a^	0.0	0^a^	0.0	3	1.6
	*Sikwekwe*	0^a^	0.0	0^a^	0.0	0^a^	0.0	2^a^	4.5	2	1.1
Treat for dermatophilosis	Yes	25^a^	41.7	2^b^	6.9	22^b^	42.3	14^b^	31.8	63	34.1
	No	13^a^	21.7	0^b^	0.0	3^a^	5.8	6^a^	13.6	22	11.9
	N/A	22^a^	36.7	27^b^	93.1	27^a^	51.9	24^a^	54.5	100	54.1
Herd problem?	Yes	35^ab^	58.3	0^c^	0.0	23^b^	44.2	32^a^	72.2	90	48.6
	No	24^ab^	40.0	25^c^	86.2	29^b^	55.8	10^a^	22.7	88	47.6
	D/K	1^a^	1.7	4^b^	13.8	0^a^	0.0	2^ab^	4.5	7	3.8
Area problem?	Yes	22^a^	36.7	1^b^	3.4	13^a^	25.0	15^a^	34.1	51	27.6
	No	35^a^	58.3	24^b^	82.8	39^ab^	75.0	26^a^	59.1	124	67.0
	D/K	3^ab^	5.0	4^b^	13.8	0^a^	0.0	3^ab^	6.8	10	5.4
Danger to humans	Yes	12^a^	20.0	0^b^	0.0	3^b^	5.8	8^a^	18.2	23	12.4
	No	41^a^	68.3	5^b^	17.2	44^c^	84.6	20^d^	45.5	110	59.5
	D/K	7^a^	11.7	24^b^	82.8	5^a^	9.6	16^c^	36.4	52	28.1

Values in the same row with the same superscript alphabet are not significantly different (*p* > 0.05). D/K = do not know; N/A, not applicable.

The farmers’ knowledge and perceptions with regard to bovine dermatophilosis were as indicated in Table 2. Most (> 50%) of the farmers from three diptanks, namely Chemawororo, Gwanyika and Koronika, had heard/were generally aware about bovine dermatophilosis, whilst at Chivero diptank less than 20% of the farmers were aware of the disease (Table 2). Of the 164 respondents who reported that they knew the local name for bovine dermatophilosis, 75.1%, 1.6% and 1.1% referred to it as *Chikundura* (Shona), Senkobo and *iSikwekwe* (isiNdebele), respectively, with the knowledge differing significantly (*p* < 0.05) across diptanks (Table 2). Across the study sites, 85 (45.9%) respondents indicated that they had knowledge regarding treatment options available for bovine dermatophilosis (Table 2); none of the respondents from Chivero diptank indicated that they had this knowledge. Tick bites were perceived by farmers as a major (45.4%) cause of bovine dermatophilosis; a proportion of farmers stated that inadequate dipping (2.2%), lashing cattle with a whip or beating them with a stick and plunge dipping (both 1.1%) contributed to the development of the disease.

At the Chemawororo and Koronika diptanks, more than 50% of the respondents stated that bovine dermatophilosis was a problem in their herds, whilst none of the farmers from Chivero diptank considered the disease a herd problem. Across the study sites, 48.6% and 27.6% of the farmers perceived dermatophilosis to be a problem in their herds and areas (geographical extent serviced by the diptank), respectively; the rest were undecided. Of the farmers interviewed, 12.4% considered bovine dermatophilosis to be a danger to humans (zoonotic). They reported that dermatophilosis caused a condition in humans manifested by skin lesions, whilst others reported that consuming meat from affected animals resulted in stomach pains.

Antibiotics belonging to the tetracycline, penicillin and gentamycin groups were reportedly used to treat bovine dermatophilosis; of these, oxytetracycline antibiotic types were the drug used by most of the farmers (79.3%) (Table 3). Stockowners said they used the drugs according to manufacturers’ instructions, with some farmers indicating that they felt that the drugs were not very effective. Fifty-two of the 63 farmers (82.6%) reported that they administered antibiotics four to seven times before the animal showed significant signs of recovery. Thirty-seven (58.5%) of the farmers treating cattle for dermatophilosis stated that it took 3–5 weeks for an animal to fully recover after treatment was initiated (Table 3). With regard to differentiating bovine dermatophilosis from diseases such as LSD and parafilariasis, 42.6% of the respondents stated that they were able to differentiate between the diseases. The respondents stated that lesions of bovine dermatophilosis differed from those of LSD in that the former disease was characterised by the formation of scabs, crusts and loss of hair on the affected part, which was not the case with LSD.

**TABLE 3:** Proportions (%) of antibiotic types used by farmers, frequency of use and apparent efficacy in treating dermatophilosis.

Antibiotic type	Number of respondents (*N* = 63)
Oxytetracycline	81.3
Penicillin	19.1
Gentamycin	1.6
**Number of times drug was administered**	-
Once	4.8
2–3 times	3.2
4–5 times	57.1
6–7 times	23.8
> 7 times	7.9
Not sure	3.2
**Days taken to heal**	-
< 7	6.3
8–14	14.3
15–21	15.9
22–28	22.0
29–35	31.7
> 35	4.8

## Discussion

The perceived prevalence of bovine dermatophilosis was relatively high (45.1%) across study sites. This prevalence was comparable to the seasonal peak of 40% from Sanyathi communal lands, Kadoma district as reported by Chatikobo *et al*. (2004). Sambo *et al*. (2007), Dalis *et al*. (2009) and Admassu and Alemu (2011) reported lower prevalences of 9.7%, 8.07% and 1.04% from Zaria, Zaria and Jos, and Ethiopia, respectively. A more targeted investigation of cattle with skin lesions in Nigeria yielded a bovine dermatophilosis prevalence of 79.1% (Dalis *et al*. 2010). The lower prevalence in the other studies could be because of the diagnostic approach used; researchers in the studies defined dermatophilosis cases as those animals that were clinically and laboratory positive. Nath *et al*. (2010) reported that laboratory-based case definitions of bovine dermatophilosis resulted in low prevalence because the *D. congolensis* bacterium was not easy to culture and isolate. The other reason for the differences could be that the study sites differed ecologically and geographically. The general awareness of bovine dermatophilosis as a cattle disease was widespread, as 79.5% of the respondents were aware of the disease. The reason for this could be because of the presence of extension services in the areas. The DLVS has veterinary extension assistants (VEAs) who are stationed within the communities that they serve. VEAs are para-veterinary professionals based at animal health management centres in communal areas (Katsande, More & Bock 2001). These VEAs are responsible for providing regular extension services in the form of farmer training, and through this, farmers are advised of diseases that occur in that particular area; they also supervise dipping in the small-holder sector. The compulsory dipping programme that is subsidised and provided by the government could also contribute to this high level of general awareness. At least once a month, cattle belonging to different livestock owners congregate at particular diptanks, providing an opportunity for farmers to interact and share knowledge about diseases. The level of awareness differed amongst the diptanks. It was high (98.3%) at Chemawororo and low (6.9%) at Chivero diptanks. This was in agreement with the findings of Munyeme *et al*. (2010), who reported that awareness of bovine tuberculosis was higher in those settings with a higher prevalence of the disease than those where a lower prevalence predominates. Lack of or lower awareness levels can contribute to the spread of a disease, in this case dermatophilosis, to new areas, with subsequent grave socio-economic implications (Bekele *et al*. 2011). To further emphasise the general awareness about the disease, a large proportion (77.8%) of the farmers indicated that there were local names for bovine dermatophilosis. Knowledge of a local name for dermatophilosis was an indication that farmers have had a long relationship with the disease. The main Shona name *Chikundura* is loosely translated to mean a disease that removes the hairs from the skin of a diseased animal, as does the isiNdebele name iSikwekwe, and farmers stated dermatophilosis differed especially from LSD as a result of the characteristic formation of scabs, crusts and loss of hair. The terms in Shona and isiNdebele can refer to a number of skin conditions, but respondents stated that in their case it referred to bovine dermatophilosis.

To evaluate the depth of the level of awareness/knowledge about bovine dermatophilosis, farmers were interviewed on their specific knowledge with regard to the causes and treatments of the disease. Across the study sites, 95 (51.4%) and 85 (45.9%) farmers stated that they had knowledge of the causes and treatment of the disease. The level of awareness or knowledge about specific issues related to the disease was substantially lower than the general level of awareness, which was 79.5%. Mosalagae, Pfukenyi and Matope (2010) reported a similar trend-that is, a decrease in knowledge when specific issues were asked about zoonotic diseases. General awareness by commercial dairy farmers was higher (80.0%) than their knowledge of specific zoonoses, such as brucellosis (40%), tuberculosis (25%) and anthrax (35%). A similar trend was observed by Tebug *et al*. (2015); in the study, farmers’ general knowledge about zoonoses was higher (30.1%) than their specific knowledge about the means of transmission of the zoonoses (6.8%). With regard to zoonoses of pet animals, Pfukenyi *et al*. (2010) reported that pet owners had a higher (77%) general awareness of pet zoonoses than their awareness and knowledge of specific zoonoses such as helminths (21.3%) and toxoplasmosis (2.1%), the latter an important zoonosis associated with cats. Whilst the quoted studies related to different diseases and dermatophilosis is a minor zoonosis (Moriello 2013), the trends in farmer knowledge are nonetheless relevant to this study.

Specific knowledge about dermatophilosis could also be limited to those farmers with cattle that experienced infections, because they are more likely to look for further information about the disease. Farmers perceived that ticks, lashing and dipping predisposed cattle to dermatophilosis, in agreement with the studies by Blood and Radostits (1989) and Hirsh *et al*. (2004), who stated that biting arthropods, trauma and wetting of skin created portals of entry for *D. congolensis*. The findings of this study indicated that animal health service providers must not only be satisfied with the attainment of a high level of general awareness about a disease; they should go further in implementing interventions that ensure that specific knowledge on treatment and causes of diseases are comparable to the level of general awareness. Specific knowledge can be increased through regular farmer training programmes.

According to the *Animal Health Act* 19:01 of Zimbabwe, bovine dermatophilosis is a notifiable disease and two SI are used to facilitate its control (Government of Zimbabwe 2010a, 2010b). For these regulations to be implemented successfully, farmers must consider the disease to be important to them. The current study revealed that across the study sites, 48.6% of the farmers considered bovine dermatophilosis to be an important disease at the herd rather than at the area level; the proportion was as high as 58.3% and 72.2% at the Chemawororo and Koronika diptanks, respectively. This could have a positive influence on efforts by the DLVS to mobilise farmers in the control of dermatophilosis, because farmers who perceived the disease to be an important constraint to their livelihoods would have a vested interest in participating in control activities.

A small proportion of farmers (12.4%) perceived that bovine dermatophilosis was a zoonotic disease, and Hirsh *et al*. (2004) and Moriello (2013) asserted that indeed the disease is a zoonosis, with lesions appearing on the hands and arms of people handling infected animals. Human infections with *D. congolensis* have been reported from the United States of America (Burd *et al*. 2007); in the report, infection was characterised by erythematous papules and pustules on the thigh. Burd *et al*. (2007) stated that in humans there was a wide clinical spectrum as a result of infection. Amor *et al*. (2011) reported a human case of dermatophilosis from Spain. It has been postulated that transmission from animals to humans was by mechanical transfer through direct contact with infected animals or debris from such animals (Amor *et al*. 2011; Burd *et al*. 2007).

The low level of awareness by farmers that dermatophilosis was a zoonotic disease was consistent with findings by Chikerema, Matope and Pfukenyi (2013), Pfukenyi *et al*.(2010) and Tebug *et al*. (2014), who reported low levels of awareness by farmers regarding certain zoonoses such as toxoplasmosis (2.1%), bovine cysticercosis (3.1%) and brucellosis (2.9%), respectively. The low level of awareness of these zoonoses could be area specific. The zoonotic potential of dermatophilosis, although not widely reported (Amor *et al*. 2011; Burd *et al*. 2007), could be used as an incentive for farmers to be involved in the control of the disease; furthermore, extension workers should make farmers aware that care is needed when handling infected animals. Currently, there are no reported cases of dermatophilosis in humans from Zimbabwe.

Farmers (34.1%) treated their cattle in the event of a disease occurrence suspected to be dermatophilosis; this was consistent with reports by Peeling and Holden (2004) that there was widespread use of drugs by producers on the basis of clinical signs without the necessary advice from trained personnel. Sirdar *et al*. (2012) also reported widespread use of antibiotics by traditional farmers to treat endemic diseases in poultry and other animals. The antimicrobial types (tetracyclines, penicillins and gentamycin) used by stockowners to control dermatophilosis were consistent with those advocated for use elsewhere (Awad *et al*. 2008; Blood & Radostits 1989; Hamid & Musa 2009; Hirsh *et al*. 2004). Tetracyclines were the drugs most widely used because in Zimbabwe they are over-the-counter drugs and they are cheaper than penicillin and gentamycin, which are prescription drugs.

The widespread use of tetracyclines and penicillins was consistent with findings by Adesokan *et al*. (2015). Adesokan *et al*. (2013) had earlier reported that there was widespread use of these drugs in African countries. Stockowners stated that they used these drugs according to the manufacturer’s instructions, for example, a single dose given once a day for 3–5 days for the short-acting formulations. Blood and Radostits (1989) advocated the use of higher doses of penicillin, which might not be as recommended by the manufacturer. For tetracyclines, Awad *et al*. (2008) recommended a double dose a day apart. These off-label recommendations mean that farmers were likely using tetracycline and penicillin drugs at lower doses at which their efficacy in the treatment of bovine dermatophilosis might not be optimal, resulting in only partial recovery of infected animals.

The challenges farmers faced in the use of the antibiotics were reflected by the high proportion of farmers who administered drugs multiple times before an animal showed signs of recovery. Hamid and Musa (2009) reported relapses in some cattle that were treated with penicillin- and tetracycline-based drugs. Burd *et al*. (2007) stated that the efficacy of parenteral antibiotics was compromised because of failure of drugs to reach organisms in the avascular upper layer of the epidermis, whilst topical medicines on the other hand were unable to reach organisms in the deep layers of the epidermis.

The fact that some farmers stated that they were able to differentiate between bovine dermatophilosis and diseases such as LSD could be as a result of the long history of association with the disease and the training that farmers received from VEAs. Peeling and Holden (2004) reported that farmers adequately trained at the community level were competent to clinically diagnose certain diseases. Chatikobo *et al*. (2013) reported that farmers from the Gokwe and Sanyathi communal lands of Zimbabwe were able to identify the common diseases that affected their herds. The ability to make a correct presumptive diagnosis is important as it has a bearing on the drugs that will be administered and the success of such treatment.

## Conclusion

This study had some limitations, some of which were the fact that a few diptanks were surveyed and the case definition for dermatophilosis was based only on clinical signs. Nevertheless, findings from this study give animal health policy makers in Zimbabwe an idea of the status of farmers’ level of awareness with regard to important aspects of bovine dermatophilosis. Knowledge of these farmers’ attributes can be harnessed by policy makers to develop community-based disease control options and treatment protocols for bovine dermatophilosis. It was noted that whilst general awareness about bovine dermatophilosis was high, knowledge on specific issues about the disease such as treatment options and zoonotic potential was low. To address this disparity in awareness, the DLVS through its extension services should capacitate farmers through targeted training on dermatophilosis and other important endemic livestock diseases. The zoonotic risk of bovine dermatophilosis to small-holder farmers in the face of high animal disease prevalence needs to be investigated. In the meantime, farmers and other animal handlers should exercise caution when handling cattle with suspect or confirmed bovine dermatophilosis.
